# Cognitive decrements in 1991 Gulf War veterans: associations with Gulf War illness and neurotoxicant exposures in the Boston Biorepository, Recruitment, and Integrative Network (BBRAIN) cohorts

**DOI:** 10.1186/s12940-023-01018-2

**Published:** 2023-10-04

**Authors:** D. Keating, M. Krengel, J. Dugas, R. Toomey, L. Chao, L. Steele, Lloyd P. Janulewicz, T. Heeren, E. Quinn, N. Klimas, K. Sullivan

**Affiliations:** 1 Department of Environmental Health, Boston University School of Public Health, 715 Albany Street, T4W, Boston, MA 02118 USA; 2 Department of Neurology, Boston University School of Medicine, 72 East Concord St, Boston, MA 02118 USA; 3 Department of Biostatistics, Boston University School of Public Health, 715 Albany St, Boston, MA 02118 USA; 4 Department of Psychological and Brain Sciences, College of Arts and Sciences, Boston University, 900 Commonwealth Ave, Boston, MA USA; 5 San Francisco Veterans Affairs Health Care System, University of California, San Francisco, CA 94143 USA; 6 Baylor College of Medicine Neuropsychiatry Division, Department of Psychiatry and Behavioral Sciences, Houston, TX 77030 USA; 7 Dr. Kiran C. Patel College of Osteopathic Medicine, Institute for Neuroimmune Medicine, Nova Southeastern University, Fort Lauderdale, FL 33314 USA; 8 Geriatric Research Education and Clinical Center, Miami VA Medical Center, Miami, FL 33125 USA

**Keywords:** Gulf war illness, Neurotoxicant, Veterans, Gulf war, common data elements, repository, cognitive

## Abstract

**Background:**

During deployment, veterans of the 1991 Gulf War (GW) were exposed to multiple war-related toxicants. Roughly a third of these veterans continue to exhibit neurotoxicant induced symptoms of Gulf War Illness (GWI), a multi-faceted condition that includes fatigue, pain and cognitive decrements. When studied empirically, both deployed veterans with exposures and those who meet the criteria for GWI are more likely to show deficits in the area of neuropsychological functioning. Although studies have shown cognitive impairments in small sample sizes, it is necessary to revisit these findings with larger samples and newer cohorts to see if other areas of deficit emerge with more power to detect such differences. A group of researchers and clinicians with expertise in the area of GWI have identified common data elements (CDE) for use in research samples to compare data sets. At the same time, a subgroup of researchers created a new repository to share these cognitive data and biospecimens within the GWI research community.

**Methods:**

The present study aimed to compare cognitive measures of attention, executive functioning, and verbal memory in a large sample of GWI cases and healthy GW veteran controls using neuropsychological tests recommended in the CDEs. We additionally subdivided samples based on the specific neurotoxicant exposures related to cognitive deficits and compared exposed versus non-exposed veterans regardless of case criteria status. The total sample utilized cognitive testing outcomes from the newly collated Boston, Biorepository, Recruitment, and Integrative Network (BBRAIN) for GWI.

**Results:**

Participants included 411 GW veterans, 312 GWI (cases) and 99 healthy veterans (controls). Veterans with GWI showed significantly poorer attention, executive functioning, learning, and short-and-long term verbal memory than those without GWI. Further, GW veterans with exposures to acetylcholinesterase inhibiting pesticides and nerve gas agents, had worse performance on executive function tasks. Veterans with exposure to oil well fires had worse performance on verbal memory and those with pyridostigmine bromide anti-nerve gas pill exposures had better verbal memory and worse performance on an attention task compared to unexposed veterans.

**Conclusions:**

This study replicates prior results regarding the utility of the currently recommended CDEs in determining impairments in cognitive functioning in veterans with GWI in a new widely-available repository cohort and provides further evidence of cognitive decrements in GW veterans related to war-related neurotoxicant exposures.

**Supplementary Information:**

The online version contains supplementary material available at 10.1186/s12940-023-01018-2.

## Background

One third of veterans who served in the 1990–1991 Gulf War (GW) have experienced chronic health symptoms including debilitating fatigue, chronic pain, and cognitive impairments [1–5]. More than thirty years after the war, veterans are still suffering from these health consequences classified as Gulf War Illness (GWI). Cognitive impairment has been noted as one of the most distressing symptoms reported by GW veterans since shortly after the war [6, 7]. The two most widely used GWI case definitions to classify the condition, Centers for Disease Control (CDC) and Kansas, both include neurocognitive impairment as one of the categories and it remains one of the most commonly reported symptom [6, 8, 9]. Prior individual studies of neuropsychological outcomes and meta-analyses of cognitive outcomes have shown cognitive decrements in veterans with GWI when compared to healthy GW veterans in the domains of attention and executive functioning, learning and memory and visuospatial functions but specific test outcomes within the domains varied by study [5, 10]. Further, environmental exposures including pesticides, pyridostigmine bromide (PB) anti-nerve gas pills and sarin nerve agent during the GW have been correlated with neuropsychological decrements, including reduced processing speed, attention and memory functioning [3, 4, 11–16]. For the past three decades, GWI researchers have attempted to gain a clearer picture of the association between toxicant exposures during the war and neurocognitive decrements in veterans with GWI, regardless of exposure status and in those exposed regardless of GWI case status. Several studies with smaller sample sizes showed neuropsychological deficits in exposed veterans, compared to unexposed veterans, while other studies clearly lacked the power to assess even moderate differences between groups [5, 10]. Even for those with adequate sample sizes in individual cohorts, results were not always consistent [5, 10]. In addition, studies were not consistent in the cognitive test measures that were used making it difficult to compare and contrast specific areas of impairment across studies. This also hindered the ability to choose the most sensitive and specific cognitive measures for use in GWI biomarker and treatment development studies. The major concern was that if the primary outcome measures were not sensitive to GWI, then it would be almost impossible to assess biomarker and treatment study outcomes appropriately. Consensus-driven common data elements (CDEs) for cognitive outcomes spearheaded by the GWI programs of the United States Department of Veteran Affairs and the Department of Defense Congressionally Directed Medical Research Program (CDMRP) were designed to encourage collaboration, data sharing and far-reaching analysis. These CDEs included tests from the cognitive domains of attention, executive function, memory, language, visuospatial, motor and mood and included 16 individuals test measures [17]. Leaders in the fields of neuropsychology and cognitive science provided expertise to create the CDEs for GWI neuropsychological testing but until now they remained to be replicated in larger study samples from different geographically represented cohorts [17]. Therefore, it was necessary to compare exposures and cognitive outcomes in a larger, more geographically representative sample with adequate power to detect differences [14]. The Boston Biorepository, Recruitment, and Integrative Network (BBRAIN) for Gulf War Illness was designed to collect new and existing data sets that could be combined and shared for future analyses. One main aim was to provide larger combined datasets for confirmation of the utility of the CDEs [18]. The current analyses were performed to replicate previous findings in a larger cohort and to further examine the relationship between toxicant exposures and neurocognitive effects in ailing GW veterans.

Data were shared from individual studies where standard cognitive testing measures including qualitative and quantitative outcomes (error types, time to task completion etc.) recommended in the GWI CDEs were used as outcomes. New and previously published BBRAIN cognitive data were combined to produce larger datasets to provide the power needed to assess the currently recommended GWI CDEs for cognitive outcomes [5, 17]. This analysis is one of the first to utilize and replicate these cognitive CDEs on a large scale to share and interpret important GWI findings. As CDEs are working recommendations, it is necessary to replicate or amend outcome measures as new evidence emerges on their current sensitivity. In addition, this combined data study sought to examine and replicate prior reported differences in neuropsychological outcomes in relation to toxicant exposures during the war with larger more representative study samples of GW veterans from the BBRAIN repository.

## Methods

### Study participants

Retrospective cognitive data from the BBRAIN repository was compiled from the Gulf War Illness Consortium (GWIC) study (*n* = 269) with 223 GWI cases and 46 controls and from participants of the University of California San Francisco and San Francisco VA (SFVA) cohorts (*n* = 142) with 89 cases and 53 controls [15, 16, 19]. These cohorts have been previously described and have been added to the BBRAIN repository for use in future studies [15, 16, 19]. The GWIC cognitive findings have not been previously published and provides new data for comparison of CDE outcomes. The SFVA cohort cognitive data have been previously published [15, 16, 19]. Total participants included 411 GW veterans, who were deployed to the Persian Gulf Theater between August 1990 and July 1991, including 312 with GWI and 99 healthy GW veteran controls as measured by the Kansas criteria [8]. All participants signed informed consents to share data for future studies (CDMRP/GWIRP GW170055, IRB # H-37,828).

### Case status

The Kansas Symptom Criteria was utilized to determine GWI case status as recommended in the GWI Common Data Elements [8, 17]. Participants were categorized as GWI cases if they endorsed multiple mild or moderate-to-severe chronic symptoms in at least three of the six statistically defined symptom domains: fatigue/sleep problems, somatic pain, neurological/cognitive/mood symptoms, gastrointestinal symptoms, respiratory symptoms, and skin abnormalities. Veterans were excluded from the study if they reported a diagnosis included in the Kansas Exclusionary Criteria. Exclusionary diagnoses included uncontrolled diabetes, heart disease other than hypertension, stroke, lupus, multiple sclerosis, cancer in the previous three years, liver disease, kidney disease, or chronic infection [8]. Veterans were also excluded if they reported a diagnosis of schizophrenia or bipolar disorder or have been hospitalized in the past two years for alcohol/drug dependence, depression, or PTSD. GW deployed veterans who did not meet case criteria or exclusionary criteria served as controls.

### Cognitive common data elements and current neuropsychological test battery

 CDEs include criteria and specific tests recommended to standardize and systematically obtain, and utilize shared data across the GWI research community [17]. A working group of GWI stakeholders including researchers, clinicians and veteran advocates met to determine a consensus list of reliable instruments for GWI research. These recommendended CDEs were determined in 2019 with the understanding that they may need to be updated as GWI research progresses. As part of the CDE process, specific CDEs were recommended for use in neuropsychological studies of GWI. Tests from each cognitive domain were chosen by a working group of GWI neuropsychological experts based on the criteria that each test had shown significant differences in at least three prior studies [17]. Each domain resulted in multiple tests that appeared sensitive and specific enough to be recommended in future biomarker and treatment studies. The CDE recommendations included tests of attention, executive function, memory, and processing speed as described in Table 1 [5].

**Table 1:** Cognitive common data elements recommendations for Gulf War illness research

Cognitive Domain	Neuropsychological Test
Premorbid Function	Word Reading Subtest of the Wide Range Achievement Test (WRAT-4)
Attention	Continuous Performance Test − 3 (CPT3)
	Wechsler Adult Intelligence Scale-IV (WAIS-IV) Recommended tests: Digit Spans
Executive Function	Delis-Kaplan Executive Function System (D-KEFS) Recommended modules: Color-Word-Interference Test, Trail Making Test, Verbal Fluency
	Wechsler Adult Intelligence Scale-IV (WAIS-IV) Recommended tests: Digit Spans
	Continuous Performance Test − 3 (CPT3)
Verbal Memory	California Verbal Learning Test - Second Edition (CVLT-II)
	Hopkins Verbal Learning Test (HVLT)
Visual Memory	Rey-Osterrieth Complex Figure Test (ROCF)
	Brief Visual Memory Test (BVMT)
Motor Function	Finger Tapping Test
	Grooved Pegboard Test
Mood	Profile of Mood States (POMS)
	Davidson Trauma Scale (DTS) - PTSD
	PTSD Checklist for DSM-V (PCL-5)
	Center for Epidemiological Studies Depression Scale (CES-D)
	Clinician Administered PTSD Scale (CAPS-5)
	Structured Clinical Interview for DSM-V (SCID-5)

From the recommended CDE list, the following neuropsychological test variables used for analyses in the current study were selected based on commonality from multiple studies within the BBRAIN repository and on sensitivity found in clinical examinations of our prior GWI cohorts (Table 2). Overlapping cognitive tests from the neuropsychological batteries in the two cohorts included the California Verbal Learning Test Second Edition (CVLT-II) [20]. Delis Kaplan Executive Function System Color-Word Interference Test (D-KEFS) [21], Trail Making Test (TMT) [22], and the Conners Continuous Performance Test Third Edition (CPT3) [23]. All tests were administered by a trained neuropsychological test administrator. All cognitive measures compared in this study are included in the current list of CDEs for GWI as reported by Cohen et al. and Jeffrey et al. and reported in Tables 1 and 2 [5, 17].

**Table 2:** BBRAIN repository neuropsychological tests by cognitive domain

Neuropsychological Test	Task	Outcome
**I. Attention/Processing Speed**		
Conner’s Continuous Performance Test Third Edition (CPT3) [23]	Target letter embedded in series of distractors; to assess sustained attention and reaction time	Reaction Time (seconds) Total Omission Errors (T score) Commission Errors (T score)
D-KEFS Color-Word Interference Test [21]: Trial 1 Trial 2	Trials 1 and 2 measure processing speed of verbal (words) and nonverbal (colors) stimuli via a timed response	Total time (seconds) Self-corrected errors (number of errors) Uncorrected errors (number of errors)
Trail Making Test [22]: Trails A	Timed connect-a-dot task to assess attention and motor control requiring number sequencing	Time to Completion (seconds)
**II. Verbal Memory**		
California Verbal Learning Test: Second Edition [20]	List of 16 nouns from 4 categories presented over multiple learning trials with recall after interference; assesses memory and learning strategies	Learning Trials 1–5 (number correct) Short Delay Recall (number correct) Long Delay Recall (number correct)
**III. Executive Functioning**		
Trail-making Test [22]: Trails B	Timed connect-a-dot task to assess alternating sequences of letters and numbers	Time to Completion (seconds)
D-KEFS Color-Word Interference [21] Test: Trial 3 Trial 4	Trials 3 and 4 measure inhibition and inhibition switching	Total time (seconds) Self-corrected errors (number of errors) Uncorrected errors (number of errors)

### War-related exposures

Environmental exposures were collected from the Kansas Gulf War Experiences and Exposures Questionnaire, a self-reported survey about exposures to chemical weapons, pesticides, and anti-nerve gas pills during the 1991 Gulf War which is part of the current CDE for GWI exposures [8, 17]. Chemical weapon exposure was determined by reporting hearing chemical alarms, while seeing smoke from oil well fires was used to determine particulate matter exposure. Pesticide exposure was determined if participants reported using pesticide cream or spray on their skin or seeing the area in which they lived sprayed or fogged with pesticides. Anti-nerve gas pill exposure was determined if the veteran reported taking pyridostigmine bromide (PB) pills [3]. For analysis purposes, GW veterans were categorized as having a respective exposure if they reported seven or more days of experiencing the area in which they lived fogged with pesticides, seeing smoke from oil well fires, hearing chemical alarms and taking PB pills. They were categorized as unexposed for these exposures if they reported less than seven days of the exposure. These exposure duration periods were based on previous research. Wolfe et al., showed that at least 7 days of exposure to 3 blister packs of PB pills per day were associated with worsened health symptoms compared to those who took less than 21 PB pills [24]. Exposure to pesticide cream on skin was a proxy for DEET exposure and was categorized as exposed if the veteran reported 31 or more days of use and unexposed if they reported less than 31 days of use. This exposure definition was based on prior exposure modeling from the RAND report and the DOD Environmental Exposure Report – Pesticides which showed that 31 or more days of exposures was the 50th percentile of exposure for DEET [25, 26]. In addition, our prior studies with pesticide applicators from the Gulf War who reported 31 or more days of exposure to pesticide cream on skin were more likely to have poorer cognitive functioning [3].

Toxicant exposures during the Gulf War were classified into exposed and unexposed for chemical weapons, oil well fires, pesticide fog and PB pills: 0–6 days of reported exposure (unexposed) and 7 or more days of reported exposure (exposed). Pesticide cream on skin was classified into unexposed if the veteran reported less than 31 days of exposure and exposed if they reported 31 or more days of exposure.

### Statistical analyses

Demographic characteristics of cases and controls are described through means and standard deviations (SD) and compared through the Wilcoxon rank sum test for continuous variables (age, years of education), and described through n’s and percentages and compared through chi-square tests for categorical variables (sex, race/ethnicity, study site). Specifically, these variables were dichotomized as male or female, White non-Hispanic or Other race/ethnicity and GWIC or UCSF study sites. Further analyses controlled for sex, race/ethicity and study site as variables of a-priori interest, and potential confounding variables including age and education identified as differing between cases and controls. While groups were compared on employment status, unemployment could in part be a consequence of GWI and so occupation (unemployment) was not considered as a potential confounder. Differences in cognitive outcomes between cases and controls were investigated through regression models, controlling for potential confounding variables of age, sex, race/ethnicity, study site and education, in order to estimate adjusted means, standard errors of the mean (SE), 95% confidence intervals (CI), and β-coefficients. Associations between environmental exposures and cognitive outcomes were also investigated through multiple linear regression models controlling for sex, age, education, study site and other exposures. These analyses were conducted for exposed vs. unexposed regardless of GWI status. These analyses were also repeated on the subset of GWI cases, and the subset of GW veteran controls for comparison purposes (Additional file 1: Appendix A and B). We performed all analyses using SAS 9.4 (Cary, NC).

## Results

### Demographics

Demographics of the combined study sample are presented in Table 3. Of the 411 study participants, 312 met criteria for GWI case status and 99 were considered controls. The study population was comprised of mostly white, non-Hispanic males approximately 52 years old with some schooling post-high school. The study population included 16% women and 19% non-white participants. The differences in age, occupation, and years of education between cases and controls were found to be statistically significant (*p* < 0.01). Overall, controls were slightly older, more highly educated, and less likely to be unemployed than GWI cases (Table 3).

**Table 3:** Descriptive table of demographics of the sample

		Overall (*N* = 411)	Cases (*N* = 312)	Control (*N* = 99)
		Mean (SD)		
Age*	---	52.6 (6.4)	52.0 (6.1)	54.7 (7.1)
		N (%)		
Race	Black/African American	44 (11.3%)	37 (12.5%)	7 (7.4%)
	White/Caucasian	317 (81.3%)	235 (79.7%)	82 (86.3%)
	Asian/Pacific Islander	3 (0.8%)	2 (0.7%)	1 (1.1%)
	Aleutian Eskimo, or American Indian	3 (0.8%)	3 (1.0%)	0 (0.0%)
	Other/Multiracial	23 (5.9%)	18 (6.1%)	5 (5.3%)
Sex	Male	344 (83.7%)	257 (82.4%)	87 (87.9%)
	Female	67 (16.3%)	55 (17.6%)	12 (12.1%)
Hispanic or Latino ethnicity	Yes	22 (8.4%)	20 (9.2%)	2 (4.3%)
	No	241 (91.6%)	197 (90.8%)	44 (95.7%)
Employment Status*	disabled/unemployed for health reasons	10 (2.5%)	9 (3.0%)	1 (1.0%)
	employed (part-time or full-time)	304 (75.4%)	226 (74.3%)	78 (78.8%)
	retired	25 (6.2%)	12 (3.9%)	13 (13.1%)
	student	6 (1.5%)	4 (1.3%)	2 (2.0%)
	unemployed/seeking employment	58 (14.4%)	53 (17.4%)	5 (5.1%)
Current marital status	single	24 (6.0%)	15 (4.9%)	9 (9.1%)
	married/living with significant other	284 (70.5%)	209 (68.8%)	75 (75.8%)
	divorced/separated	89 (22.1%)	74 (24.3%)	15 (15.2%)
	widowed	6 (1.5%)	6 (2.0%)	0 (0.0%)
Years of education*				
	Mean years of education (SD)	15.4 (2.3)	14.5 (2.3)	16.3 (2.5)
Branch of Service	Army	255 (62.04%)	195 (62.5%)	60 (60.6%)
	Air Force	32 (7.79%)	23 (7.4%)	9 (9.1%)
	Marines	64 (15.57%)	50 (16.0%)	14 (14.2%)
	Navy	42 (10.22%)	26 (9.3%)	16 (16.2%)

*p < 0.01

### Gulf War illness cases status and cognitive outcomes

**Fig. 1:**
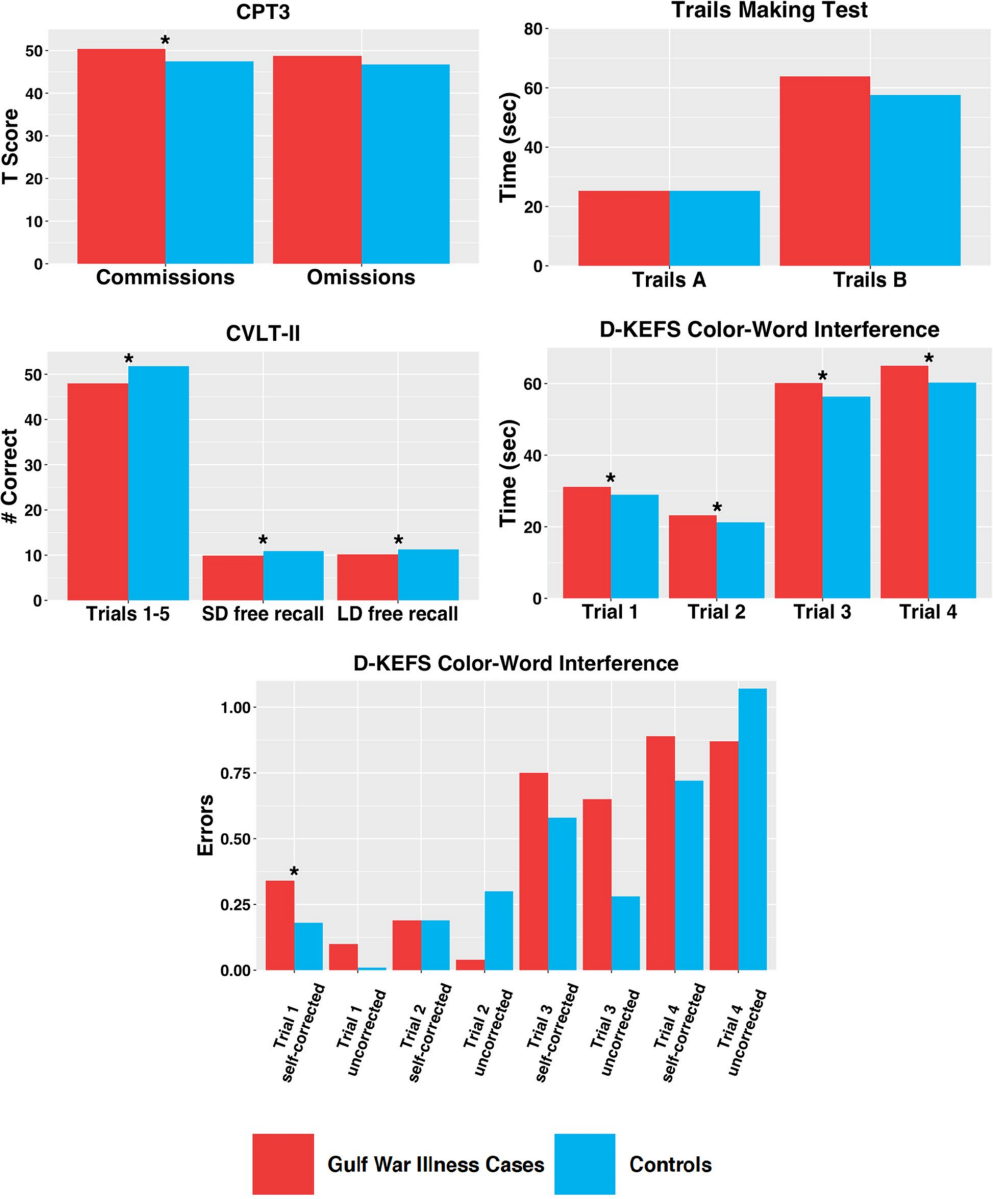
Neuropsychological mean outcome measures by Gulf War Illness status

After adjusting for age, gender, race/ethnicity, study site and years of education, veterans with GWI had significantly slower D-KEFS Color-word times on all trials compared to controls (*p* < 0.05). In addition, significantly higher mean T-scores on the Conners CPT3 commission errors were found in GWI cases compared to controls (*p* < 0.05). GWI cases also had significantly fewer words recalled in CVLT-II learning trials 1–5, and short-and-long-delayed recall than controls (*p* < 0.05, Fig. 1; Table 4). Findings suggest differences in the attention, executive and verbal memory domains.

**Table 4:** Neuropsychological measures by Gulf War illness status

			GWI Cases (*n* = 312)			Controls (*n* = 99)			p-value
		Unadjusted Mean (SD)	Adjusted Mean (SE)	95% CI	Unadjusted Mean (SD)	Adjusted Mean (SE)	95% CI	β	
*Attention/processing speed*	CPT 3 Omissions	49.76(10.07)	48.56(0.76)	47.07, 50.05	47.27(9.36)	46.73(1.15)	44.46, 48.99	1.83	0.1349
	CPT 3 Commissions*	53.38(10.52)	50.37(0.77)	48.85, 51.88	47.78(9.93)	47.45(1.17)	45.15, 49.75	2.92	0.0193
	CPT 3 Hit Reaction Time Raw Score	421.44(81.76)	425.09(6.32)	412.66, 437.52	431.81(74.97)	425.56(9.60)	406.68, 444.45	-0.48	0.9628
	D-KEFS trial 1 time (sec)*	32.6(7.82)	31.15(0.55)	30.08, 32.22	29.67(6.55)	28.98(0.85)	27.32, 30.65	2.17	0.0151
	D-KEFS trial 1 self-corrected errors*	0.36(0.71)	0.34(0.05)	0.25, 0.44	0.17(0.41)	0.18(0.08)	0.04, 0.33	0.16	0.0427
	D-KEFS trial 1 uncorrected errors	0.16(0.58)	0.10(0.04)	0.02, 0.18	0.07(0.46)	0.01(0.06)	-0.12, 0.14	0.09	0.1944
	D-KEFS trial 2 time (sec)*	24.17(5.65)	23.21(0.39)	22.43, 23.98	21.57(4.40)	21.19(0.61)	20.00, 22.39	2.01	0.0017
	D-KEFS trial 2 self-corrected errors	0.17(0.45)	0.19(0.03)	0.12, 0.25	0.17(0.43)	0.19(0.05)	0.09, 0.29	-0.01	0.9266
	D-KEFS trial 2 uncorrected errors	0.05(0.26)	0.04(0.19)	-0.33, 0.41	0.52(5.03)	0.30(0.29)	-0.27, 0.88	-0.26	0.3890
	TMT Trail A: Time (sec)	32.00(13.58)	25.30(0.98)	23.36, 27.25	27.46(10.02)	25.21(1.30)	22.63, 27.79	0.09	0.9494
*Verbal Memory*	CVLT-II Correct in Trials 1–5*	46.43(10.28)	47.97(0.73)	46.53, 49.41	50.52(9.79)	51.77(1.13)	49.54, 54.00	-3.8	0.0015
	CVLT-II Correct in short delay free recall*	9.39(3.28)	9.88(0.23)	9.42, 10.33	10.56(3.04)	10.89(0.36)	10.18, 11.60	-1.0	0.0077
	CVLT-II Correct in long delay free recall*	9.72(3.41)	10.22(0.25)	9.73, 10.70	10.93(3.17)	11.27(0.38)	10.51, 12.02	-1.1	0.0095
*Executive Functioning*	D-KEFS trial 3 time (sec)*	62.01(16.23)	60.11(1.14)	57.88, 62.35	57.71(14.88)	56.34(1.76)	52.88, 59.81	3.77	0.0420
	D-KEFS trial 3 self-corrected errors	0.82(1.39)	0.75(0.10)	0.56, 0.94	0.59(1.00)	0.58(0.15)	0.28, 0.87	0.18	0.2696
	D-KEFS trail 3 uncorrected errors	0.75(3.14)	0.65(0.21)	0.24, 1.07	0.37(1.13)	0.28(0.33)	-0.37, 0.93	0.37	0.2817
	D-KEFS trial 4 time (sec)*	67.53(19.31)	65.02(1.35)	62.37, 67.67	60.68(15.80)	60.24(2.09)	56.13, 64.35	4.78	0.0300
	D-KEFS trial 4 self-corrected errors	0.85(1.13)	0.89(0.08)	0.73, 1.04	0.63(0.85)	0.72(0.12)	0.47, 0.96	0.17	0.1995
	D-KEFS trial 4 uncorrected errors	1.06(1.77)	0.87(0.22)	0.44, 1.31	1.14(5.05)	1.07(0.34)	0.39, 1.74	-0.20	0.5868
	TMT Trail B: Time (sec)	73.50(29.44)	63.88(3.32)	57.32, 70.44	64.74(31.64)	57.55(4.40)	48.84, 66.25	6.33	0.2007

Multiple Logistic Regression modeling was used for analysis in the table adjusting for age, gender, years of education, race/ethnicity and study site. Beta is the regression parameter representing the difference in adjusted means. p-value testing whether beta = 0, or equivalently a difference in adjusted means. Abbreviations: SD (standard deviation), SE (standard error of the mean), CI (confidence interval). CIs are shown for adjusted means

*p < 0.05

**p < 0.001

### Environmental exposures and cognitive outcomes

Multiple linear regression modeling was used to compare mean neuropsychological measures in the unexposed versus exposed groups. All analyses were adjusted for age, gender, years of education, study site and the other exposures listed (Table 5, Additional file 1: Appendix A). For example, hearing chemical alarms was controlled for seeing smoke from oil well fires, using pesticide cream or spray on skin, seeing the area in which you lived fogged or sprayed with pesticides, and taking PB pills.

### Chemical weapons

Among all veterans, exposure to chemical weapons was significantly associated with slower times on the Trail Making Test Trails  B (*p* < 0.005, Table 5) suggesting differences in the executive domain. When performing the same analysis among only veterans with GWI, exposed GWI veterans had significantly slower times to completion on Trail Making Test Trails B (*p* < 0.005, Additional file 1: Appendix A). When performing the same analyses among only GW veteran controls, exposed veterans had significantly higher Conners CPT3 omisson scores and slower times on the Trail Making Test Trails B and slower speed on the D-KEFS Color-Word Interference Trial 3 (*p* < 0.05, Additional file 1: Appendix B).

### Smoke from oil well fires

Exposure to smoke from oil well fires was significantly associated with fewer correct words in both the CVLT-II short and long delay recall in all GW veterans (*p* < 0.05, Table 5) suggesting differences in verbal memory domain. Among GWI cases, those exposed to smoke from oil well fires had significantly fewer words recalled on the CVLT-II short delay recall and slower times on the D-KEFS Color Word Interference trial 2 (*p* < 0.05, Additional file 1: Appendix A). Among GW controls, those exposed to smoke from oil well fires had no significant differences when compared with those with no oil well fire exposures.

### Pesticide exposure

In all GW veterans, reporting using pesticide cream or spray on skin (a proxy for DEET exposure) was not significantly associated with any cognitive differences. In GWI cases only, there were no significant associations found between exposure and neuropsychological test scores, (Additional file 1: Appendix A). Among GW controls, total time on D-KEFS trial 3 and time to completion on the Conners CPT3 were significantly slower with pesticide cream or spray exposure (Additional file 1: Appendix B). All GW veterans who reported seeing the area in which they lived fogged or sprayed with pesticides (proxy for organophosphate and carbamate pesticides) for more than 7 days had significantly slower time on the D-KEFS Color-Word Interference trial 1 and more self-corrected errors on trial 4 (*p* < 0.05, Table 5) suggesting attention and executive functioning differences. Among those with GWI, the same measures were found to significantly differ between groups (*p* < 0.05, Additional file 1: Appendix A). Among GW controls, those exposed to pesticide sprays and fogs, had significantly more errors on the D-KEFS Color-Word Interference Trial 3 and slower time on Trail Making Test Trails B (*p* < 0.05, Additional file 1: Appendix B).

### PB anti-nerve gas pills

In all GW veterans, exposure to PB pills was significantly associated with more self-corrected errors on D-KEFS Color Word trial 1 and more words recalled in the CVLT-II learning trials 1–5 as well as more words recalled on the short-and-long delay recall (*p* < 0.05, Table 5) suggesting mixed differences in attention and memory domains. Among only those with GWI, exposed veterans showed the same pattern of significant differences as the overall GW veteran group (*p* < 0.05, Additional file 1: Appendix A). Among GW controls, exposed veterans had no significant differences compared with the unexposed group (Additional file 1: Appendix B).

**Table 5:** Neuropsychological measures by toxicant exposure in all GW veterans controlling for age, gender, years of education, site and other exposures

			Exposed	Unexposed		
			Adjusted Mean		β	*p*-value
			*N* = 152	*N* = 214		
**Chemical Weapons (sarin/cyclosarin)**	*Attention/processing speed*	CPT 3 Omissions	48.03	48.59	0.57	0.604
		CPT 3 Commissions	50.30	48.41	-1.89	0.092
		CPT 3 Hit Reaction Time Raw Score	420.24	434.92	14.68	0.111
		D-KEFS trial 1 time (sec)	31.17	31.11	-0.05	0.948
		D-KEFS trial 1 self-corrected errors	0.31	0.37	0.06	0.384
		D-KEFS trial 2 time (sec)	23.10	22.44	-0.66	0.251
		D-KEFS trial 2 self-corrected errors	0.14	0.18	0.04	0.458
		TMT Trail A: Time (sec)	26.80	25.91	-0.90	0.562
	*Verbal Memory*	CVLT-II Correct in Trials 1–5	47.83	49.06	1.23	0.247
		CVLT-II Correct in short delay free recall	10.04	10.19	0.15	0.659
		CVLT-II Correct in long delay free recall	10.14	10.76	0.62	0.083
	*Executive Functioning*	D-KEFS trial 3 time (sec)	61.17	57.96	-3.21	0.053
		D-KEFS trial 3 self-corrected errors	0.77	0.65	-0.12	0.390
		D-KEFS trial 4 time (sec)	65.78	62.92	-2.86	0.152
		D-KEFS trail 4 self-corrected errors	0.95	0.94	-0.01	0.915
		TMT Trail B: Time (sec)*	72.72	59.14	-13.60	0.009
			*N* = 229	*N* = 137		
**Smoke from oil well fires**	*Attention/processing speed*	CPT3 Omissions	48.81	47.80	-1.01	0.348
		CPT3 Commissions	49.99	48.72	-1.27	0.250
		CPT3 Hit Reaction Time Raw Score	429.33	425.83	-3.49	0.700
		D-KEFS trial 1 time (sec)	31.73	30.55	-1.18	0.140
		D-KEFS trial 1 self-corrected errors	0.36	0.33	-0.03	0.642
		D-KEFS trial 2 time (sec)*	23.40	22.14	-1.27	0.028
		D-KEFS trial 2 self-corrected errors	0.18	0.14	-0.04	0.473
		TMT Trail A: Time (sec)	26.64	26.07	-0.57	0.704
	*Verbal Memory*	CVLT-II Correct in Trials 1–5	47.49	49.41	1.92	0.072
		CVLT-II Correct in short delay recall*	9.64	10.58	0.94	0.005
		CVLT-II Correct in long delay recall*	10.10	10.81	0.71	0.049
	*Executive Functioning*	D-KEFS trial 3 time (sec)	60.42	58.70	-1.72	0.301
		D-KEFS trial 3 self-corrected errors	0.77	0.65	-0.12	0.481
		D-KEFS trial 4 time (sec)	65.54	63.16	-2.39	0.234
		D-KEFS trail 4 self-corrected errors	1.04	0.85	-0.19	0.107
		TMT Trail B: Time (sec)	64.12	67.74	3.62	0.465
			*N* = 150	*N* = 216		
**Pesticide cream or spray on skin (DEET)**	*Attention/processing speed*	CPT3 Omissions	48.43	48.19	-0.23	0.834
		CPT3 Commissions	48.78	49.93	1.15	0.317
		CPT3 Hit Reaction Time Raw Score	432.61	422.55	-10.1	0.286
		D-KEFS trial 1 time (sec)	31.58	30.71	-0.87	0.297
		D-KEFS trial 1 self-corrected errors	0.38	0.30	-0.08	0.262
		D-KEFS trial 2 time (sec)	22.76	22.78	0.02	0.977
		D-KEFS trial 2 self-corrected errors	0.12	0.20	0.08	0.122
		TMT Trail A: Time (sec)	27.22	25.49	-1.74	0.276
	*Verbal Memory*	CVLT-II Correct in Trials 1–5	47.83	49.07	1.24	0.260
		CVLT-II Correct in short delay recall	9.89	10.34	0.46	0.189
		CVLT-II Correct in long delay recall	10.41	10.49	0.08	0.830
	*Executive Functioning*	D-KEFS trial 3 time (sec)	59.71	59.41	-0.30	0.192
		D-KEFS trial 3 self-corrected errors	0.68	0.74	0.06	0.670
		D-KEFS trial 4 time (sec)	63.66	65.04	1.38	0.507
		D-KEFS trail 4 self-corrected errors	0.88	1.01	0.13	0.276
		TMT Trail B: Time (sec)	69.06	62.80	-6.25	0.240
			*N* = 68	*N* = 298		
**Pesticide fog (organophosphate/ carbamate)**	*Attention/processing speed*	CPT3 Omissions	49.00	47.62	-1.38	0.310
		CPT3 Commissions	49.39	49.32	-0.07	0.962
		CPT3 Hit Reaction Time Raw Score	430.57	424.59	-5.97	0.601
		D-KEFS trial 1 time (sec)*	32.15	30.13	-2.02	0.046
		D-KEFS trial 1 self-corrected errors	0.39	0.29	-0.10	0.279
		D-KEFS trial 2 time (sec)	23.06	22.45	-0.65	0.375
		D-KEFS trial 2 self-corrected errors	0.13	0.19	0.05	0.409
		TMT Trail A: Time (sec)	27.34	25.38	-1.96	0.375
	*Verbal Memory*	CVLT-II Correct in Trials 1–5	47.93	48.96	1.03	0.443
		CVLT-II Correct in short delay recall	10.02	10.20	0.18	0.671
		CVLT-II Correct in long delay recall	10.36	10.55	0.18	0.683
	*Executive Functioning*	D-KEFS trial 3 time (sec)	60.68	58.44	-2.25	0.283
		D-KEFS trial 3 self-corrected errors	0.72	0.70	-0.02	0.924
		D-KEFS trial 4 time (sec)	65.36	63.34	-2.02	0.423
		D-KEFS trial 4 self-corrected errors *	1.14	0.75	-0.39	0.008
		TMT Trail B: Time (sec)	66.51	65.35	-1.15	0.875
			*N* = 184	*N* = 182		
**PB Pills**	*Attention/processing speed*	CPT3 Omissions	48.36	48.26	-0.11	0.921
		CPT3 Commissions	48.88	49.83	0.95	0.390
		CPT3 Hit Reaction time raw score	428.62	426.54	-2.08	0.819
		D-KEFS trial 1 time (sec)	31.21	31.07	-0.14	0.858
		D-KEFS trial 1 self-corrected errors*	0.42	0.27	-0.15	0.036
		D-KEFS trial 2 time (sec)	22.73	22.81	0.08	0.889
		D-KEFS trial 2 self-corrected errors	0.19	0.13	-0.07	0.187
		TMT Trail A: Time (sec)	25.64	27.08	1.44	0.350
	*Verbal Memory*	CVLT-II Correct in Trials 1–5*	49.69	47.21	-2.47	0.023
		CVLT-II Correct in short delay recall*	10.58	9.65	-0.92	0.006
		CVLT-II Correct in long delay recall*	10.97	9.94	-1.03	0.004
	*Executive Functioning*	D-KEFS trial 3 time (sec)	59.55	59.57	0.02	0.992
		D-KEFS trial 3 self-corrected errors	0.72	0.69	-0.03	0.845
		D-KEFS trial 4 time (sec)	63.97	64.73	0.705	0.442
		D-KEFS trail 4 self-corrected errors	0.95	0.94	-0.01	0.909
		TMT Trail B: Time (sec)	62.78	69.08	6.30	0.217

*p < 0.05

Multiple Regression analysis was used for analysis in the table adjusting for age, gender, education, study site and other exposures

## Discussion

Neuropsychological tests are proxies for central nervous system (CNS) function; it has long been known that GW veterans have had difficulty with CNS and specifically cognitive functioning since their return from deployment [2, 3, 5, 10, 11, 14–16]. However, different neuropsychological tests were used in prior studies making comparisons across studies challenging. This is particularly important now that substantial progress has been made with regard to biomarker and treatment development for GWI and using cognitive outcomes measures that differentiate cases of the disorder are critical to comparatively assess study outcomes. An initial approach to deal with this problem was a meta-analysis of cognitive outcomes used in the GWI field and from this cognitive common data elements (CDEs) were recommended for the field based on these findings [5, 10]. Several differences in neuropsychological function have been observed between GW veterans and healthy controls and have also been associated with neurotoxicant exposures during the war [5]. A meta-analysis of neuropsychological characteristics of GWI published in 2017 identified significantly decreased performance in the functional domains of executive function, visuospatial skills, and learning and memory across 16 studies [10]. High mixed exposure to pesticides and PB anti-nerve gas pills has also been associated with significantly slowed information processing speed, increased attentional errors, poor visual memory functioning, and increased mood complaints [3]. Conversely, PB pill usage without high pesticide exposure during the war associated with better verbal memory functioning [3]. The cognitive CDEs that were recommended for the GWI field in 2019 by a working group of experts including VA and DOD investigators included 16 tests across 7 cognitive domains (attention, executive function, language, memory, visuospatial, motor and mood) [17]. However, these CDEs still remained to be evaluated across multiple GW veteran cohorts representing different parts of the country and branches of service in studies that were conducted more recently to replicate findings from prior studies. This study assessed cognitive CDE outcomes in a newly combined cohort of veterans from the BBRAIN repository. The BBRAIN repository is being shared for multiple ongoing and planned studies of GWI and therefore, ensuring the reproduciblity of prior findings in the field within this new combined cohort is essential to establishing the feasibility of use of the repository for future biomarker and treatment development studies. This is particularly revelant because BBRAIN includes previously unpublished as well as prior published cognitive testing results. Specifically, our current results showed differences in measures of attention, processing speed, verbal memory and executive functioning in veterans with GWI compared to those without the disorder. These results replicate the findings of our prior meta-analysis of 16 publications from studies that had been conducted between 1992 and 2015, where potential confounders could not be controlled for at the individual level [10]. The current study was able to control for multiple potential confounders and still replicated many of the prior meta-analytic findings therefore strengthening the body of evidence supporting utility of these specific cognitive test measures in GWI research. These results suggest that CPT, Trail Making Test, CVLT and D-KEFS Color Word Interference test should be used in future studies of GWI. We have found that not only summary scores but also error types and qualitative outcome scores also differ among cases and controls and among exposed vs. unexposed groups. Our recommendation is therefore, to utilize tests and specific test outcomes as listed in Table 2 for future biomarker and treatment studies of veterans with GWI.

In addition, we showed neurotoxicant exposures including chemical weapons, pesticides, PB pills and oil well fires are associated with cognitive decrements in attention, executive function and verbal memory. Although no clear patterns appeared with regard to cognitive test outcomes and exposures, it is worth noting that exposures related to acetylcholisterase inhibition (chemical weapons, pesticide creams, pesticide sprays/fogs) were associated with executive system decrements as noted on Trails B time to completion, D-KEFS Color-Word Interference Test Trial 3 and Trial 1 time to completion and Trial 4 increased self-corrected errors. These results do correspond with executive system function decrements reported in other pesticide and sarin exposed groups [3, 13, 14]. In addition, smoke from oil well fires was associated with poorer verbal memory recall on CVLT-II. To our knowledge, this is the first time that verbal memory has been associated with smoke from oil wells in GWI. In addition, exposure to oil well smoke was recently associated with increased risk of GWI in APOE4 carrier veterans [27]. Conversely, PB alone was associated with better performance on verbal memory outcomes. This replicates prior reports of better verbal memory from a different cohort of GW veterans reporting high PB exposure but few other exposures during the war [5]. Therefore, this combined data study from the BBRAIN repository network now reproduces these CDEs for cognitive outcomes for GWI and adds to the literature for those with neurotoxicant exposures during the war.

Specifically, it was found that veterans who met criteria for GWI relative to healthy veteran controls without GWI showed impairments in the domains of attention and memory with short-and-long delayed recall on a verbal list learning task on the CVLT-II suggesting clear differences between cases and controls. Further, GW veterans who reported neurotoxicant exposures showed deficits in cognitive domains relative to those without such exposures regardless of case status. However, exposure-cognitive associations in the full combined cohort appeared to be largely driven by associations in the GWI cases. A number of associations were identified only in controls, however, suggesting possible CNS effects of GW exposures that are not limited to veterans who meet criteria for GWI. Specifically, cognitive testing results suggested a relationship between exposure to chemical weapons (sarin/cyclosarin) and diminished executive control and slower processing speed on a task of alternating letter and number sequencing. Exposure to smoke from oil well fires was significantly associated with poorer immediate and sustained verbal memory. Skin pesticide exposure was used as a proxy for DEET exposure during the war and was significantly associated with poor executive control and slower processing speed on two measures in GW controls only. While pesticide sprays (organophosphate and carbamates) was associated with attention and executive function on a test of inhibition switching. It should be noted that DEET concentration used during this deployment was up to 75% active ingredient, much higher than current standards. In addition, exposure to anti-nerve gas (PB) pills appeared to be associated with both protective and detrimental effects in relation to attention and memory outcomes. PB pill usage was associated with more self-corrected errors on the D-KEFS Color Word Intereference test compared with unexposed veterans. Use of PB pills had strong relationships with memory functioning specifically in verbal learning as well as verbal long and short delay measures and was protective. This corresponds with prior studies with preventive health military personnel with only PB exposure [3]. However, this work also reported that exposure to both PB and pesticides was detrimental to verbal memory outcomes. When these environmental exposures were examined among veterans who met the definition for GWI case status, the cognitive outcomes differed slightly and had somewhat fewer differences but the results generally indicate that the exposure-cognitive associations are largely driven by associations in GWI cases. There were however, associations identified only in controls suggesting possible CNS effects of GW exposures that are not limited to veterans with GWI. This suggests that exposures including to acetylcholinesterase (AChE) inhibitors and oil well fires regardless of case status may also be important to monitor with regard to chronic cognitive outcomes but more confirmatory research is needed in this area.

This study documents and replicates reduced performance on tasks of sustained and divided attention, executive tasks including impulsivity and inattentiveness and verbal learning and recall memory in veterans with GWI from the currently recommended CDEs for cognitive outcomes [17]. It also documents the association of these decrements with environmental toxicant exposures during the war. Replicating these CDE recommended tests for future studies in current GW veteran cohorts including the now widely available BBRAIN repository is critical for determining which tests will be sensitive for use in ongoing biomarker and treatment studies within the field. It is important to note verbal memory decrements as this may indicate the need for future follow up as these veterans continue to age and may be more vulnerable to neurodegenerative disorders if they have GWI and/or prior neurotoxicant exposures [12, 28]. It is also important to note that not all neurotoxicant exposures have resulted in the same cognitive decrements even when they may have been in the same chemical class (i.e. AChE inhibitors). This suggests that specific exposures to repellents or organophosphate and carbamate pesticides may have individual and combined effects on cognitive outcomes for exposed veterans [29, 30]. Future studies should utilize the cognitive CDEs supported by findings from this study and may also benefit from comparing more sensitive computerized and screening measures as well as those that assess real-world subjective impacts of cognitive changes as Gulf War veterans age [31, 32]. However, the CDE tests utilized in this report could be used as minimum data elements for all future studies evaluating cognitive outcomes in veterans with GWI.

Despite replicating many of the CDEs for neuropsychological testing, our study is limited to the most commonly used neuropsychological tests and cognitive domains evaluated for the multiple study datasets shared in the BBRAIN repository including the CVLT-II, CPT3, D-KEFS Color-Word Interference Test and Trail Making Test. Due to the heterogeneity of prior studies, it was not possible to assess all neuropsychological tests within the currently recommended CDEs. This included a lack of visuospatial tests across our repository studies. In addition, further insight into cognitive deficits may be gained with a wider array and fuller battery of tests including newer computerized and screening instruments from the CDEs [31]. Testing results from CDE measures also largely remain to be correlated with subjective cognitive complaints to assess their real-world implications [32]. Another limitation of our study as with almost all of the GW studies was measuring exposures using self-report and recall from events from many years ago. However, due to the lack of official notification of most exposures; self-report is the best way to capture exposures during the Gulf War.

These findings suggest that GW veterans, especially those who suffer from GWI, have sustained neurotoxic wounds including impaired cognition relative to controls who do not meet the Kansas criteria. This work substantiates the need for more refined exposure-based CDEs as well. As GW veterans age, neurocognitive deficits that may already exist as a result of toxic wounds have the potential to become more debilitating and overtax available cognitive reserves and potentially lead to increased risk for neurodegenerative disorders [33]. An increase in attention and care is needed for this GW veteran population to mitigate these cognitive deficits in their daily lives. Identification of the most sensitive and specific neuropsychological measure CDEs is integral for assessing treatment trial efficacy and biomarker sensitivity: two areas critical for the GWI field.

## Conclusion

This study documents and replicates reduced performance on tasks of sustained and divided attention, executive function and verbal memory in veterans with GWI from the currently recommended common data elements for cognitive outcomes [17]. It also documents the association of some of these decrements with environmental toxicant exposures during the war. Specifically, CPT3, D-KEFS Color-word Interference Test, CVLT-II and Trail Making Test should be used in future studies of veterans with GWI. It would also be helpful to compare these cognitive outcomes with subjective cognitive complaints to further document the daily impact of these decrements.

### Supplementary Information

**Additional file 1.**

## Data Availability

The dataset(s) supporting the conclusions of this article is(are) available in the BBRAIN repository, https://wwwapp.bumc.bu.edu/BEDAC_BBrainRetro.

## Abbreviations

AChE: Acetylcholinesterase
BBRAIN: Boston, Biorepository, Recruitment and Integrative Network for Gulf War Illness
CDC: Center for Disease Control
CDE: Common Data Elements
CDMRP: Congressionally Directed Medical Research Program
CNS: Central Nervous System
CPT3: Conners Performance Test Third Edition
CVLT-II: Califonia Verbal Language Test Second Edition
D-KEFS: Delis-Kaplan Executive Function System
GW: Gulf War
GWI: Gulf War Illness
PB: Pyridostigmine bromide
PTSD: Post Traumatic Stress Disorder
TMT: Trail Making Test
VA: Veterans Affairs
