# Supplementary material for: Cognitive decrements in 1991 Gulf War veterans: associations with Gulf War illness and neurotoxicant exposures in the Boston Biorepository, Recruitment, and Integrative Network (BBRAIN) cohorts
Source: Environ Health. 2023 Oct 4;22:68. doi: 10.1186/s12940-023-01018-2 (PMC10548744; doi:10.1186/s12940-023-01018-2)
Supplement: Supplementary file 1 — Additional file 1. [file 12940_2023_1018_MOESM1_ESM.docx]

**Appendix A. Neuropsychological measures by toxicant exposure in GW veterans with Gulf War Illness**

|  |  |  | **Exposed** | **Unexposed** |  |  |
| --- | --- | --- | --- | --- | --- | --- |
|  |  |  | Adjusted Mean | | β | p-value |
|  |  |  | N=130 | N=145 |  |  |
| **Chemical Weapons**  **(sarin/cyclosarin)** | *Attention/ processing speed* | CPT 3 Omissions | 47.28 | 49.33 | 2.06 | 0.105 |
|  |  | CPT 3 Commissions | 51.29 | 49.21 | -2.07 | 0.115 |
|  |  | CPT 3 Hit Reaction Time Raw Score | 411.79 | 432.07 | 20.28 | 0.061 |
|  |  | D-KEFS trial 1 time (sec) | 31.47 | 31.35 | -0.12 | 0.900 |
|  |  | D-KEFS trial 1 self-corrected errors | 0.33 | 0.44 | 0.11 | 0.211 |
|  |  | D-KEFS trial 2 time (sec) | 23.33 | 22.59 | -0.73 | 0.283 |
|  |  | D-KEFS trial 2 self-corrected errors | 0.11 | 0.20 | 0.09 | 0.126 |
|  |  | TMT Trail A: Time (sec) | 26.09 | 24.97 | -1.12 | 0.580 |
|  | *Verbal Memory* | CVLT-II Correct in Trials 1-5 | 46.69 | 48.38 | 1.69 | 0.160 |
|  |  | CVLT-II Correct in short delay free recall | 9.76 | 10.06 | 0.30 | 0.439 |
|  |  | CVLT-II Correct in long delay free recall | 9.79 | 10.55 | 0.76 | 0.067 |
|  | *Executive Functioning* | D-KEFS trial 3 time (sec) | 60.87 | 58.94 | -1.93 | 0.320 |
|  |  | D-KEFS trial 3 self-corrected errors | 0.73 | 0.61 | -0.12 | 0.495 |
|  |  | D-KEFS trial 4 time (sec) | 67.55 | 63.72 | -3.83 | 0.111 |
|  |  | D-KEFS trail 4 self-corrected errors | 0.99 | 0.96 | -0.03 | 0.857 |
|  |  | **TMT Trail B: Time (sec)*** | 75.82 | 58.96 | -16.9 | 0.010 |
|  |  |  | N=182 | N=93 |  |  |
| **Smoke from oil well fires** | *Attention/ processing speed* | CPT3 Omissions | 48.86 | 47.75 | -1.12 | 0.387 |
|  |  | CPT3 Commissions | 50.88 | 49.62 | -1.27 | 0.339 |
|  |  | CPT3 Hit Reaction Time Raw Score | 425.29 | 418.56 | -6.73 | 0.540 |
|  |  | D-KEFS trial 1 time (sec) | 32.68 | 30.68 | -1.47 | 0.137 |
|  |  | D-KEFS trial 1 self-corrected errors | 0.39 | 0.37 | -0.02 | 0.815 |
|  |  | **D-KEFS trial 2 time (sec)*** | 23.79 | 22.13 | -1.66 | 0.020 |
|  |  | D-KEFS trial 2 self-corrected errors | 0.16 | 0.14 | -0.02 | 0.720 |
|  |  | TMT Trail A: Time (sec) | 26.37 | 24.69 | -1.68 | 0.437 |
|  | *Verbal Memory* | CVLT-II Correct in Trials 1-5 | 46.70 | 48.37 | 1.67 | 0.181 |
|  |  | **CVLT-II Correct in short delay recall*** | 9.49 | 10.33 | 0.83 | 0.038 |
|  |  | CVLT-II Correct in long delay recall | 9.94 | 10.41 | 0.47 | 0.275 |
|  | *Executive Functioning* | D-KEFS trial 3 time (sec) | 61.34 | 58.47 | -2.86 | 0.157 |
|  |  | D-KEFS trial 3 self-corrected errors | 0.77 | 0.56 | -0.21 | 0.255 |
|  |  | D-KEFS trial 4 time (sec) | 66.92 | 64.35 | -2.57 | 0.306 |
|  |  | D-KEFS trail 4 self-corrected errors | 1.07 | 0.88 | -0.19 | 0.22 |
|  |  | TMT Trail B: Time (sec) | 61.72 | 73.05 | 11.34 | 0.101 |
|  |  |  | N=134 | N=141 |  |  |
| **Pesticide cream or spray on skin (DEET)** | *Attention/ processing speed* | CPT3 Omissions | 48.41 | 48.20 | -0.21 | 0.870 |
|  |  | CPT3 Commissions | 49.49 | 51.01 | 1.53 | 0.245 |
|  |  | CPT3 Hit Reaction Time Raw Score | 424.35 | 419.50 | -4.85 | 0.656 |
|  |  | D-KEFS trial 1 time (sec) | 31.46 | 31.36 | -0.10 | 0.921 |
|  |  | D-KEFS trial 1 self-corrected errors | 0.40 | 0.36 | -0.05 | 0.626 |
|  |  | D-KEFS trial 2 time (sec) | 22.58 | 23.34 | 0.76 | 0.283 |
|  |  | D-KEFS trial 2 self-corrected errors | 0.11 | 0.20 | 0.09 | 0.108 |
|  |  | TMT Trail A: Time (sec) | 27.11 | 23.94 | -3.17 | 0.124 |
|  | *Verbal Memory* | CVLT-II Correct in Trials 1-5 | 46.85 | 48.22 | 1.37 | 0.270 |
|  |  | CVLT-II Correct in short delay recall | 9.61 | 10.20 | 0.59 | 0.139 |
|  |  | CVLT-II Correct in long delay recall | 10.09 | 10.26 | 0.17 | 0.693 |
|  | *Executive Functioning* | D-KEFS trial 3 time (sec) | 58.77 | 61.03 | 2.26 | 0.258 |
|  |  | D-KEFS trial 3 self-corrected errors | 0.62 | 0.72 | 0.10 | 0.579 |
|  |  | D-KEFS trial 4 time (sec) | 64.16 | 67.11 | 2.95 | 0.232 |
|  |  | D-KEFS trail 4 self-corrected errors | 0.89 | 1.06 | 0.17 | 0.244 |
|  |  | TMT Trail B: Time (sec) | 73.28 | 61.49 | -11.8 | 0.072 |
|  |  |  | N=59 | N=216 |  |  |
| **Pesticide fog (organophosphate and carbamate)** | *Attention/ processing speed* | CPT3 Omissions | 48.72 | 47.89 | -0.83 | 0.585 |
|  |  | CPT3 Commissions | 50.41 | 50.09 | -0.32 | 0.839 |
|  |  | CPT3 Hit Reaction Time Raw Score | 422.46 | 421.40 | -1.06 | 0.934 |
|  |  | **D-KEFS trial 1 time (sec)*** | 33.05 | 30.66 | -2.39 | 0.046 |
|  |  | D-KEFS trial 1 self-corrected errors | 0.31 | 0.21 | -0.10 | 0.373 |
|  |  | D-KEFS trial 2 time (sec) | 23.26 | 22.66 | -0.60 | 0.472 |
|  |  | D-KEFS trial 2 self-corrected errors | 0.11 | 0.20 | 0.09 | 0.198 |
|  |  | TMT Trail A: Time (sec) | 25.71 | 25.35 | -0.37 | 0.892 |
|  | *Verbal Memory* | CVLT-II Correct in Trials 1-5 | 47.24 | 47.83 | 0.60 | 0.682 |
|  |  | CVLT-II Correct in short delay recall | 9.89 | 9.92 | 0.03 | 0.950 |
|  |  | CVLT-II Correct in long delay recall | 10.14 | 10.21 | 0.08 | 0.880 |
|  | *Executive Functioning* | D-KEFS trial 3 time (sec) | 61.43 | 58.38 | -3.04 | 0.196 |
|  |  | D-KEFS trial 3 self-corrected errors | 0.61 | 0.73 | 0.12 | 0.560 |
|  |  | D-KEFS trial 4 time (sec) | 66.69 | 64.58 | -2.12 | 0.467 |
|  |  | **D-KEFS trial 4 self-corrected errors*** | 1.20 | 0.75 | -0.45 | 0.009 |
|  |  | TMT Trail B: Time (sec) | 62.47 | 72.30 | 9.83 | 0.255 |
|  |  |  | N=156 | N=119 |  |  |
| **PB Pills** | *Attention/ processing speed* | CPT3 Omissions | 48.59 | 48.02 | -0.58 | 0.654 |
|  |  | CPT3 Commissions | 49.59 | 50.91 | 1.32 | 0.318 |
|  |  | CPT3 Hit Reaction time raw score | 424.15 | 419.71 | -4.44 | 0.686 |
|  |  | D-KEFS trial 1 time (sec) | 31.14 | 31.68 | 0.54 | 0.586 |
|  |  | **D-KEFS trial 1 self-corrected errors*** | 0.48 | 0.28 | -0.20 | 0.032 |
|  |  | D-KEFS trial 2 time (sec) | 22.65 | 23.27 | 0.62 | 0.383 |
|  |  | D-KEFS trial 2 self-corrected errors | 0.21 | 0.10 | -0.11 | 0.057 |
|  |  | TMT Trail A: Time (sec) | 24.44 | 26.62 | 2.17 | 0.295 |
|  | *Verbal Memory* | **CVLT-II Correct in Trials 1-5*** | 49.08 | 46.00 | -3.08 | 0.014 |
|  |  | **CVLT-II Correct in short delay recall*** | 10.41 | 9.41 | -1.00 | 0.013 |
|  |  | **CVLT-II Correct in long delay recall*** | 10.75 | 9.60 | -1.14 | 0.008 |
|  | *Executive Functioning* | D-KEFS trial 3 time (sec) | 59.74 | 60.07 | 0.33 | 0.871 |
|  |  | D-KEFS trial 3 self-corrected errors | 0.66 | 0.68 | 0.01 | 0.941 |
|  |  | D-KEFS trial 4 time (sec) | 64.45 | 66.82 | 2.38 | 0.340 |
|  |  | D-KEFS trial 4 self-corrected errors | 0.99 | 0.96 | -0.02 | 0.887 |
|  |  | TMT Trail B: Time (sec) | 63.02 | 71.75 | 8.74 | 0.184 |

*p<0.05

*p<0.05

Note: Multiple Regression was used for analysis in the table adjusting for age, gender, education, study site and other exposures.

**Appendix B. Neuropsychological measures by toxicant exposure in GW veteran controls**

|  |  |  | **Exposed** | **Unexposed** |  |  |
| --- | --- | --- | --- | --- | --- | --- |
|  |  |  | Adjusted Mean | | β | p-value |
|  |  |  | N=22 | N=69 |  |  |
| **Chemical Weapons**  **(sarin/cyclosarin)** | *Attention/ processing speed* | **CPT 3 Omissions*** | 50.38 | 45.37 | -5.00 | 0.033 |
|  |  | CPT 3 Commissions | 45.45 | 44.95 | -0.50 | 0.837 |
|  |  | CPT 3 Hit Reaction Time Raw Score | 461.70 | 458.51 | -3.19 | 0.869 |
|  |  | D-KEFS trial 1 time (sec) | 30.13 | 30.39 | 0.26 | 0.860 |
|  |  | D-KEFS trial 1 self-corrected errors | 0.26 | 0.23 | -0.03 | 0.774 |
|  |  | D-KEFS trial 2 time (sec) | 22.54 | 22.72 | 0.18 | 0.858 |
|  |  | D-KEFS trial 2 self-corrected errors | 0.29 | 0.17 | -0.12 | 0.262 |
|  |  | TMT Trail A: Time (sec) | 28.11 | 27.16 | -0.94 | 0.727 |
|  | *Verbal Memory* | CVLT-II Correct in Trials 1-5 | 53.28 | 51.07 | -2.22 | 0.336 |
|  |  | CVLT-II Correct in short delay free recall | 11.95 | 11.02 | -0.93 | 0.176 |
|  |  | CVLT-II Correct in long delay free recall | 12.36 | 12.00 | -0.36 | 0.618 |
|  | *Executive Functioning* | **D-KEFS trial 3 time (sec)*** | 65.45 | 57.92 | -7.52 | 0.023 |
|  |  | D-KEFS trial 3 self-corrected errors | 1.28 | 1.26 | -0.02 | 0.942 |
|  |  | D-KEFS trial 4 time (sec) | 60.49 | 62.01 | 1.52 | 0.671 |
|  |  | D-KEFS trail 4 self-corrected errors | 0.78 | 0.86 | 0.08 | 0.698 |
|  |  | TMT Trail B: Time (sec) | 69.64 | 64.40 | -5.24 | 0.545 |
|  |  |  | N=47 | N=44 |  |  |
| **Smoke from oil well fires** | *Attention/ processing speed* | CPT3 Omissions | 47.71 | 48.03 | 0.32 | 0.871 |
|  |  | CPT3 Commissions | 45.43 | 44.96 | -0.47 | 0.823 |
|  |  | CPT3 Hit Reaction Time Raw Score | 457.83 | 462.38 | 4.54 | 0.783 |
|  |  | D-KEFS trial 1 time (sec) | 30.40 | 30.11 | -0.29 | 0.825 |
|  |  | D-KEFS trial 1 self-corrected errors | 0.26 | 0.24 | -0.02 | 0.845 |
|  |  | D-KEFS trial 2 time (sec) | 22.65 | 22.60 | -0.05 | 0.954 |
|  |  | D-KEFS trial 2 self-corrected errors | 0.25 | 0.21 | -0.04 | 0.657 |
|  |  | TMT Trail A: Time (sec) | 27.31 | 27.96 | 0.65 | 0.779 |
|  | *Verbal Memory* | CVLT-II Correct in Trials 1-5 | 51.09 | 53.26 | 2.17 | 0.292 |
|  |  | CVLT-II Correct in short delay recall | 10.94 | 12.03 | 1.08 | 0.080 |
|  |  | CVLT-II Correct in long delay recall | 11.63 | 12.73 | 1.10 | 0.091 |
|  | *Executive Functioning* | D-KEFS trial 3 time (sec) | 60.88 | 62.50 | 1.62 | 0.577 |
|  |  | D-KEFS trial 3 self-corrected errors | 1.22 | 1.32 | 0.11 | 0.601 |
|  |  | D-KEFS trial 4 time (sec) | 61.37 | 61.12 | -0.25 | 0.938 |
|  |  | D-KEFS trail 4 self-corrected errors | 0.95 | 0.69 | -0.26 | 0.145 |
|  |  | TMT Trail B: Time (sec) | 65.76 | 68.28 | 2.52 | 0.735 |
|  |  |  | N=16 | N=75 |  |  |
| **Pesticide cream or spray on skin (DEET)** | *Attention/ processing speed* | CPT3 Omissions | 47.23 | 48.52 | 1.29 | 0.609 |
|  |  | CPT3 Commissions | 43.89 | 46.50 | 2.60 | 0.328 |
|  |  | **CPT3 Hit Reaction Time Raw Score*** | 481.13 | 439.08 | -42.0 | 0.049 |
|  |  | D-KEFS trial 1 time (sec) | 31.62 | 28.90 | -2.72 | 0.113 |
|  |  | D-KEFS trial 1 self-corrected errors | 0.34 | 0.16 | -0.18 | 0.088 |
|  |  | D-KEFS trial 2 time (sec) | 23.50 | 21.75 | -1.75 | 0.132 |
|  |  | D-KEFS trial 2 self-corrected errors | 0.21 | 0.25 | 0.04 | 0.762 |
|  |  | TMT Trail A: Time (sec) | 26.92 | 28.35 | 1.43 | 0.641 |
|  | *Verbal Memory* | CVLT-II Correct in Trials 1-5 | 53.46 | 50.88 | -2.58 | 0.331 |
|  |  | CVLT-II Correct in short delay recall | 12.06 | 10.91 | -1.16 | 0.145 |
|  |  | CVLT-II Correct in long delay recall | 12.95 | 11.42 | -1.53 | 0.068 |
|  | *Executive Functioning* | **D-KEFS trial 3 time (sec)*** | 66.14 | 57.23 | -8.91 | 0.019 |
|  |  | D-KEFS trial 3 self-corrected errors | 1.29 | 1.25 | -0.03 | 0.898 |
|  |  | D-KEFS trial 4 time (sec) | 62.07 | 60.43 | -1.64 | 0.691 |
|  |  | D-KEFS trail 4 self-corrected errors | 0.72 | 0.92 | 0.19 | 0.400 |
|  |  | TMT Trail B: Time (sec) | 63.34 | 70.71 | 7.37 | 0.453 |
|  |  |  | N=9 | N=82 |  |  |
| **Pesticide fog (organophosphate and carbamate)** | *Attention/ processing speed* | CPT3 Omissions | 48.79 | 46.96 | -1.83 | 0.582 |
|  |  | CPT3 Commissions | 44.32 | 46.08 | 1.76 | 0.615 |
|  |  | CPT3 Hit Reaction Time Raw Score | 477.74 | 442.46 | -35.3 | 0.207 |
|  |  | D-KEFS trial 1 time (sec) | 30.20 | 30.32 | 0.12 | 0.959 |
|  |  | D-KEFS trial 1 self-corrected errors | 0.29 | 0.20 | -0.09 | 0.545 |
|  |  | D-KEFS trial 2 time (sec) | 23.63 | 21.62 | -2.01 | 0.196 |
|  |  | D-KEFS trial 2 self-corrected errors | 0.29 | 0.18 | -0.11 | 0.495 |
|  |  | TMT Trail A: Time (sec) | 30.10 | 25.17 | -4.93 | 0.249 |
|  | *Verbal Memory* | CVLT-II Correct in Trials 1-5 | 50.77 | 53.58 | 2.81 | 0.429 |
|  |  | CVLT-II Correct in short delay recall | 11.24 | 11.73 | 0.48 | 0.648 |
|  |  | CVLT-II Correct in long delay recall | 12.12 | 12.24 | 0.13 | 0.910 |
|  | *Executive Functioning* | D-KEFS trial 3 time (sec) | 62.16 | 61.21 | -0.96 | 0.849 |
|  |  | **D-KEFS trial 3 self-corrected errors*** | 1.85 | 0.69 | -1.17 | 0.002 |
|  |  | D-KEFS trial 4 time (sec) | 62.99 | 59.51 | -3.48 | 0.528 |
|  |  | D-KEFS trial 4 self-corrected errors | 0.90 | 0.74 | -0.16 | 0.613 |
|  |  | **TMT Trail B: Time (sec)*** | 82.16 | 51.89 | -30.3 | 0.030 |
|  |  |  | N=28 | N=63 |  |  |
| **PB Pills** | *Attention/ processing speed* | CPT3 Omissions | 47.83 | 47.92 | 0.09 | 0.968 |
|  |  | CPT3 Commissions | 44.57 | 45.82 | 1.24 | 0.585 |
|  |  | CPT3 Hit Reaction time raw score | 463.83 | 456.38 | -7.44 | 0.681 |
|  |  | D-KEFS trial 1 time (sec) | 31.26 | 29.25 | -2.01 | 0.162 |
|  |  | D-KEFS trial 1 self-corrected errors | 0.24 | 0.25 | 0.01 | 0.915 |
|  |  | D-KEFS trial 2 time (sec) | 23.35 | 21.90 | -1.45 | 0.139 |
|  |  | D-KEFS trial 2 self-corrected errors | 0.21 | 0.25 | 0.03 | 0.745 |
|  |  | TMT Trail A: Time (sec) | 27.53 | 27.74 | 0.22 | 0.940 |
|  | *Verbal Memory* | CVLT-II Correct in Trials 1-5 | 53.79 | 50.56 | -3.23 | 0.149 |
|  |  | CVLT-II Correct in short delay recall | 12.13 | 10.84 | -1.29 | 0.055 |
|  |  | CVLT-II Correct in long delay recall | 12.77 | 11.59 | -1.17 | 0.096 |
|  | *Executive Functioning* | D-KEFS trial 3 time (sec) | 61.93 | 61.44 | -0.48 | 0.877 |
|  |  | D-KEFS trial 3 self-corrected errors | 1.28 | 1.26 | -0.03 | 0.903 |
|  |  | D-KEFS trial 4 time (sec) | 62.69 | 59.81 | -2.88 | 0.406 |
|  |  | D-KEFS trial 4 self-corrected errors | 0.70 | 0.95 | 0.25 | 0.412 |
|  |  | TMT Trail B: Time (sec) | 64.12 | 69.92 | 5.80 | 0.525 |

*p<0.05

Note: Multiple regression was used for analysis in the table adjusting for age, gender, education, study site and other exposures.
